# Evaluation of Visual Field and Balance Function Alterations in Patients Who Underwent Dermatochalasis Surgery

**DOI:** 10.1155/2020/1310947

**Published:** 2020-04-09

**Authors:** Melih Akidan, Deniz Turgut Coban, Muhammet Kazım Erol, Uğur Balci

**Affiliations:** ^1^Antalya Kepez State Hospital, Department of Ophthalmology, Antalya, Turkey; ^2^Antalya Education and Research Hospital, Department of Ophthalmology, Antalya, Turkey

## Abstract

**Purpose:**

To compare perioperative visual field (VF), balance functions (BF), and changes in the other ocular parameters in patients undergoing upper eyelid dermatochalasis (DC) surgery.

**Methods:**

One hundred and fifty-eight eyes of 79 patients who underwent DC surgery were included in the study. The VF, BF, intraocular pressure (IOP), pachymetry (PM), macular, and optic nerve measurements were recorded. Measurements were repeated at postoperative month 1. The preoperative and postoperative ocular measurements and the balance data were compared.

**Results:**

Nineteen of 79 (24.05%) patients were male and 60 of 79 (75.95%) were female, while the mean age of the patients was 58.65 ± 7.38 years. There were statistically significant differences in terms of VF and macular thickness between the preoperative and postoperative values. The improvements in mean defect, standard loss variance, and mean sensitivity values of global VF parameters in both eyes were statistically significant after surgery. Central macular thickness, mean macular thickness, and macular volume decreased significantly in all eyes after surgery (*p* < 0.05).

**Conclusions:**

Although a marked improvement was observed in VF and peripheral vision after surgery, no significant change was found in BF parameters including primarily falling risk. The significant change in the macular parameters was only remarkable, and we think that the decrease was due to subtle vasospasm. There is a need for further comprehensive studies including especially patients older than 65 with a view to understanding the effect of DC surgery on BF.

## 1. Introduction

Dermatochalasis (DC) is the most frequent cause of acquired pseudoptosis, which causes a decrease in superior visual field (VF) due to the loosening of the upper lid skin, which is folded by the atrophy of the elastic tissue with advanced age [1, 2]. It leads to difficulties in primary sight, reading, and visual functions due to VF defects [3]. In addition, patients may experience issues such as appearing constantly sleepy and having a tired look, loss of self-esteem, and even being perceived negatively in society [4]. Numerous studies have demonstrated positive changes in subjective visual functions and in quality of life along with an improvement in the objective VF after surgery [5, 6].

Posture control depends on the integration of information derived from proprioceptive, vestibular, and visual perceptive systems [7]. Visual acuity, contrast sensitivity, depth perception, and peripheral vision are key visual functions necessary to maintain physical balance [8]. It has been established that especially peripheral vision has a more prominent role in back and forth oscillation of the body when compared to the central vision [9]. Furthermore, the visual component plays a prominent role as a compensatory mechanism in postural stability in situations where there is also proprioceptive inadequacy [10]. It was stated that the risk of falling in elderly people was higher since they were not able to detect the environmental threats because of VF defects [11]. For those people having impaired vision, many vision-dependent activities and daily tasks are difficult or impossible to perform, reducing their ability to perform daily living activities and maintain independence, which has a negative impact on their quality of life [12].

To the best of our knowledge, our study is the first to measure BF, macula, and optic nerve parameters as well in addition to perioperative VF, IOP, and PM measurements in patients with dermatochalasis. In particular, we think that BF evaluation will contribute to the literature as it can improve the quality of life in addition to its effect on visual quality after surgery.

## 2. Materials and Methods

**Figure 1:**
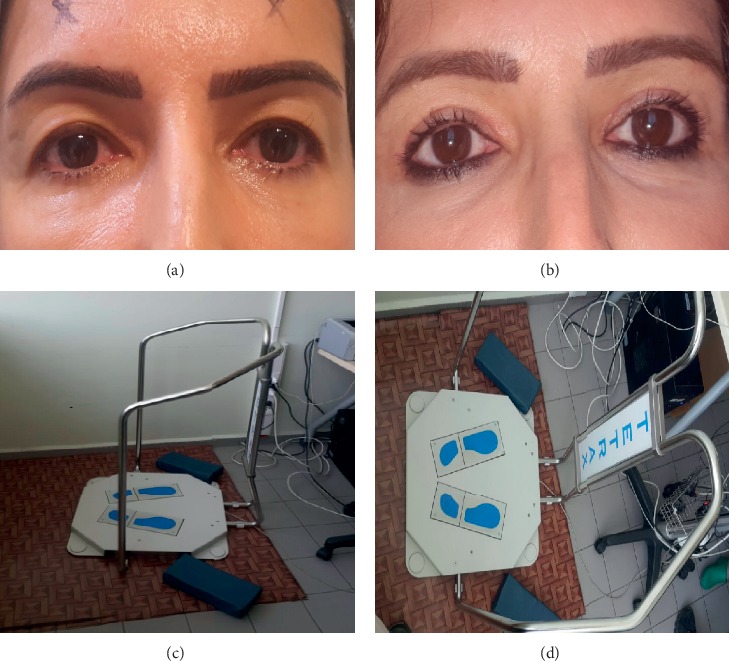
(a) Upper eyelid appearance before dermatochalasis surgery. (b) 1st month after surgery. (c) A computer-based static posturography device with balance function measurements. (d) The foot platform of the device and the foam used in the different stages of the test.

This study was performed retrospectively in Antalya Training and Research Hospital, Department of Ophthalmology, upon the protocol approval by the Institutional Review Board. Seventy-nine patients whose VF and BF were measured before and after DC surgery were included in the study **(**Figure 1). The patients who had ocular diseases related to an existing pathology, blepharoptosis associated with decreased levator function, psychiatric diseases, diseases of the vestibular system, central, or peripheral nervous systems which might affect BF and the patients who were unable to comply with the VF and static posturography measurements, unable to stand up without support or auxiliary devices, and pregnant or nursing mothers were excluded from the study.

During the surgical procedure, before the sterile prepping, incision line was drawn while the patients were in the sitting position. While drawing the upper crease line from the corner of the eye to the highest point of the midline, we made sure that the length was 7-8 mm for male patients and 9-10 mm for female patients.

For patients who were compatible with fixation reliability criteria before DC surgery and one month after the surgery, mean defect (MD), standard loss variance (sLV), and mean sensitivity (MS) values were assessed by perimetry (Octopus 900 Haag-Streit, Interzeag AG, Schlieren-Zurich, Switzerland). Central macular thickness (CMT), mean macular thickness (MMT), macular volume (MV), disc area, rim area, rim area/disc area ratio (RA/DA), ganglion cell layer (GCL), retinal nerve fiber layer (RNFL) thickness measurements, and also thickness measurements within four quadrants as superior, nasal, temporal, and inferior were assessed by optical coherence tomography (Cirrus HD-OCT 5000, Carl Zeiss Meditec Inc, Dublin, CA, USA). Intraocular pressure (IOP) and pachymetry (PM) values were measured with tonometry (Canon Noncontact TX-20).

BF was measured by computer-based static posturography (Tetrax, Sunlight Medical Ltd.). Posturography is a diagnostic system which analyzes a subject's balance and the mechanisms employed to maintain balance. This method of posturography is based on the assessment of the vertical pressure fluctuations on four independent force plates, each placed beneath the two heels and toe parts of the subject while he/she stands in an upright position. The Tetrax plates (dimensions: length 25 cm, width 13 cm, and height 8 cm each) are equipped with a strain gauge, the output of which consists of fluctuations of voltage. This output is transformed by an A-D device into a digital signal, which is analyzed by Tetrax software. The weight of the examinee is automatically controlled by the software while height does not interfere with the Tetrax parameters, as shown by systemic examinations [13]. Four basic parameters, which were the stability index (STI), Fourier Harmony Index (FHI), weight percentage and weight distribution index (WDI), heel to toe for the feet, and pressure patterns of left and right foot synchronization, were measured by the Tetrax device in 8 different positions and frequencies, respectively. These 8 different positions are as follows: NO (normal open position: standing straight with eyes open), NC (normal closed position: standing straight with eyes closed), PO (pillows open: standing on pillows, with eyes open), PC (pillows closed: standing on pillows, with eyes closed), HR (head right: standing with the head turned right and eyes closed), HL (head left: standing with the head turned left and eyes closed), HB (head back: standing with tilted backward at a 30-degree angle, with eyes closed), HF (head forward: standing with head tilted forward about 30 degrees, with eyes closed), and the measurement time for each position is 32 seconds.

STI is the numeric variable expression of a patient's postural defects and controls, which cannot be detected clinically. It evaluates changes in the center of gravity. The total amount of sway from the four footplates (right and left heels, right and left toes) is summed up and then divided by the subject's weight (the amplitude of the indices of postural sway is affected by vertical pressure; therefore, the division of the postural sway indices by the subject's weight is used in the posturographic methodology to cancel out the positive correlation of weight to the amplitude). That parameter was calculated as the square root of the sum of squared differences between adjacent pressure fluctuation signals, transmitted by the A-D device and sampled at a rate of 32 Hz for each of the four platforms. Limit values are considered as the standard deviations from 1.5 to 3. Higher values indicate higher imbalance [14, 15].

The FHI is a regression analysis of postural sway intensity through the Fourier transform, which shows a different frequency for each lesion that causes instability. Transformations consisting of four independent wave signals are divided into eight different frequencies and recorded. The FHI evaluates the regression pattern of the eight Tetrax frequency bands. Tetrax program compares the Fourier power values of posturographic performance to a mathematically computed regression curve and evaluates the discrepancy between the graph obtained from the collected data and the theoretical “ideal” regression in the form of a coefficient. This spectral pattern is designated as Fourier Harmony and its coefficient as the Fourier Harmony Index. FHI is the assessment of normal posture performance. Values from 0.9 to 0.99 are the normal limits. If lower values are detected, they indicate problems in the visual, vestibular, and postural feedback mechanism [16].

WDI shows discordant weight distribution in the foot platform and may be an indication of an orthopedic problem. WDI assesses the synchronization pressure patterns of feet and the pressure on the plates where the heel and the toe are placed, while the effectiveness of coordinated movements between the heel and the toes of each foot are also evaluated.

Falling index is used to reevaluate the data. Its algorithm is based on the addition of standard deviation scores, which are obtained when calculating by how many standard deviations the performance of an examinee deviate from the mean of the normative database provided by IBS software. Adding the standard scores for stability, Fourier intensities of ∼0.3 and ∼1.00 Hz, and synchronizations, a fall index is graded as minimum falling risk (0–36), moderate level (37–58), high level (58–100), according to which precautions and supportive treatment can be planned [8, 17].

## 3. Statistical Analysis

All data were analyzed with SPSS (Statistical Package for the Social Sciences) software for Windows (v22.0; IBM, Armonk, NY, USA). Individual and aggregate data were summarized using descriptive statistics including mean, standard deviations, medians (min-max), frequency distributions, and percentages. Dependent variables with normal distribution were compared with Student's *t*-test for paired samples. For the continuous variables that were not normally distributed, the Wilcoxon test was used to compare the preoperative and postoperative values. *p* values <0.05 were considered statistically significant.

## 4. Results

Out of 79 patients (158 eyes) who underwent upper DC surgery (blepharoplasty), 19 (24.05%) were male and 60 (75.95%) were female, while the mean age of the patients was 58.65 ± 7.38 years (Ranged: 36–84 years). In our study, no significant difference was found between the preoperative and postoperative visual acuity, IOP, and PM for both eyes (Table 1).

When the VF global indicator results were compared; preoperative values of MD and sLV were found to decrease significantly compared to the values of postoperative MD and sLV for both eyes (*p* < 0.05) (Table 1). Furthermore, preoperative values of MS measured were found to increase significantly after the operation for both eyes (*p* < 0.05) (Table 1).

According to macular thickness and volume evaluation results; a statistically significant (*p* < 0.05) decrease was observed in CMT, MMT, and MV values measured in all eyes (Table 2). There were no statistically significant differences in terms of optic nerve head parameters, RNFL, and GCL thickness between the preoperative and postoperative values in both eyes (Table 2).

While the balance functions in STI, FHI, and WDI dimensions were measured in various body, head, and eye positions (Table 3), falling risk slightly decreased after the operation; however, no statistically significant difference was found (Table 3).

## 5. Discussion

The VF test is applied for legitimizing the blepharoplasty. According to the international standard guidelines, upper margin-reflect distance should be more than 2.5 mm, and the VF has to improve by more than 30% after blepharoplasty [18]. Kosmin et al. observed a significant improvement after blepharoplasty where they used similar VF global indexes in their study [19]. However, the objective improvement in the visual field of patients alone cannot completely indicate the subjective perception of visual quality by patients, changes in visual functions, and increased quality of life. For that reason, studies evaluating quality of vision and life have been reported. Nevertheless, the VF test is an important parameter as the visual acuity test which is used for disability scaling. Even the changes in VF of patients may reflect the limitations in the quality of life and thus, have been used as a legal criterion for reimbursement [20]. Zinkernagel et al. reported that vision change might occur as changes in corneal astigmatism develop in patients with advanced dermatochalasis during corneal topography and periopertive evaluation [21]. Kim et al. pointed to the improvement in contrast sensitivity and visual quality after blepharoplasty [22]. Federici et al. evaluated ptosis cases by the margin distance, VF, and life quality questionnaire postoperatively to assess patients' subjective state and found that the existing entities had a high correlation [23]. The findings of our study demonstrated that the decrease in VF might be improved by blepharoplasty; therefore, in the light of studies conducted before, it might enable a significant improvement in the quality of vision and life for the patients with DC.

Thirty percent of the population aged 65 and older die or become permanently disabled due to falling once a year or more [24]. Every 10% loss in visual field corresponds to an 8% higher risk of falls in adults older than 65 years [25]. Cahill et al. emphasized that loss of peripheral vision was more highly associated with falls than visual acuity, contrast sensitivity, stereo acuity, and central visual field loss, and superior visual field loss was just as important as inferior field loss [26]. Moreover, Luna et al. demonstrated that the differences in lower and upper visual field loss in patients with glaucoma did not constitute balance functions differences [27]. On the other hand, refraction disorders such as uncorrected astigmatism and conditions affecting visual acuity, visual field, and contrast sensitivity like cataract also affect postural stability [28, 29]. In our study, we thought that improved viral field and the directly associated improvements increased postural stability and could improve quality of life. However, although there was a marked improvement in visual field, no significant change was observed in fall risk contrary to the abovementioned literature. The values of falling risk were within the moderate risk range; thus, perioperative decrease is worth mentioning even though it was not found to be statistically significant. We might not notice the change especially above the age of 65 due to the large age range of the patients included in our study (mean: 58.65 ± 7.38, ranged: 36–84 years).

Retinal changes are observed mainly at early stages with the rise of retrobulbar hemorrhage after the blepharoplasty, followed by the application of local anesthetics containing epinephrine, vasospasm, inflammation, and systemic hypotension, respectively [30, 31]. Theoretically it could be caused by pulling on the fat pedicles or by the use of vasopressor agents such as adrenaline in the local anesthetic infiltrates or by the action of vasoactive agents released from extravasated blood [32]. Çalık et al. reported that iatrogenic cystoid macular edema and papillitis decreased spontaneously at week 5 after surgery [33]. Transient visual loss after transconjunctival lower lid blepharoplasty was reported, which was considered to be associated with vascular spasm in retina or optic nerve circulation [31]. Macular thickness alterations are also observed after the strabismus surgery. Mintz et al. found an increase in macular thickness after strabismus surgery due to the mechanical effect of the new arrangement of extraocular muscles, postoperative inflammation, and the alteration in the blood-retinal barrier [34]. Similar to the findings in the literature, macula thickness was usually reported to be increased after extraocular surgeries such as both blepharoplasty and strabismus. In our study, however, although perioperative complications were observed, a statistically significant decrease was found in the macular parameters, which might be related to subtle vasospasm rather than inflammation.

Atalay et al. assessed corneal hysteresis, corneal resistance factor, PM, and IOP and reported no significant difference in PM and IOP values after DC surgery [35]. Bleyen et al. documented an angle closure glaucoma which they attributed to infiltrative anesthesia, constrictive bandage, and patients' anxiety after blepharoplasty surgery [36]. There was no statistically significant difference in IOP and PM values in our study. In the published data, there is no research found regarding the comparison of optic nerve parameters after upper lid surgeries, including ptosis. However, similar causes affecting macula may also impair the optic nerve circulation. Moreover, no statistically significant difference was found in RNFL and GCL measurements, along with data regarding the optic nerve parameters in our study. Furthermore, we did not detect any additional optic nerve or retinal disease until the IOP alteration and measurement time. We assume that this was due to the fact that no serious complications were observed in our patients.

In conclusion, BF, macula, and optic sinir parameters were evaluated for the first time in our study in addition to perioperative VF, IOP, and PM measurements in patients with dermatochalasis. Although a marked improvement was found in peripheral vision with VF, no significant change was observed in general in BF parameters including primarily fall risk. The significant change in the macular parameters was only remarkable, and we think that the decrease was due to subtle vasospasm. There is a need for further comprehensive studies including especially patients older than 65 with a view to better understanding the effect of blepharoplasty surgery on fall risk and balance functions.

## Figures and Tables

**Table 1:** Perioperative comparison of visual acuity, pachymetry, intraocular pressure, and visual field values of all eyes.

Parameters	Before	After	*p* value
Visual acuity	0.970 ± 0.095	0.970 ± 0.095	1.000^b^
PM	547.29 ± 26.631	545.93 ± 27.085	0.270^a^
IOP	15.562 ± 2.629	15.543 ± 2.673	0.876^b^
MD	9.515 ± 4.815	4.423 ± 3.495	0.001^*∗*^^b^
MS	17.394 ± 4.883	22.408 ± 3.500	0.001^*∗*^^b^
sLV	6.041 ± 2.303	3.294 ± 1.679	0.001^*∗*^^b^

^a^Student's *t*-test for paired samples; ^b^Wilcoxon test; ^*∗*^Statistically significant (*p* < 0.05). PM, pachymetry; IOP, intraocular pressure; MD, mean deviation. MS, mean sensivity; sLv, standart loss variance.

**Table 2:** Perioperative comparison of macular and optic disc parameters of all eyes.

Parameters	Before	After	*p* value
CMT	249.33 ± 20.425	247.12 ± 28.368	0.043^*∗*^^b^
MMT	281.62 ± 14.849	278.27 ± 18.736	0.025^*∗*^^b^
MV	10.141 ± 0.533	10.012 ± 0.670	0.008^*∗*^^b^
RNFL	94.07 ± 9.542	93.58 ± 11.069	0.271^b^
Superior	116.72 ± 18.545	114.16 ± 18.365	0.558^b^
Nasal	71.15 ± 10.993	72.26 ± 10.782	0.893^b^
Temporal	64.77 ± 11.446	64.08 ± 12.816	0.120^a^
Inferior	123.82 ± 15.928	122.53 ± 19.102	0.174^b^
Disc area	1.903 ± 0.314	1.911 ± 0.291	0.523^b^
Rim area	1.427 ± 0.272	1.435 ± 0.275	0.958^b^
RA/DA	0.457 ± 0.163	0.465 ± 0.161	0.134^b^
GCL	83.34 ± 7.283	82.39 ± 9.227	0.155^b^

^a^Student's *t*-test for paired samples; ^b^Wilcoxon test; ^*∗*^Statistically significant (*p* < 0.05). CMT, central macular thickness; MMT, mean macular thickness; MV, macular volume. RNFL, retinal nerve fiber layer; RA/DA, rim area disc/area ratio; GCL, gangliyon cell layer.

**Table 3:** Perioperative comparison of balance function measurements.

Parameters	Before	After	*p* value
Fall index	50 ± 26.980	48.76 ± 29.894	0.889^b^
*NO (normal open position)*			
FHI	0.85 ± 0.144	1.77 ± 8.515	0.340^b^
WDI	6.29 ± 3.465	6.31 ± 3.515	0.974^b^
STI	21.23 ± 11.717	23.93 ± 20.561	0.638^b^
*NC (normal closed position)*			
FHI	0.76 ± 0.174	0.81 ± 0.183	0.037^b^
WDI	6.36 ± 3.635	6.15 ± 3.324	0.685^b^
STI	20.55 ± 9.639	22.21 ± 10.555	0.512^b^
*PO (pillows open position)*			
FHI	0.879 ± 0.146	0.862 ± 0.139	0.082^b^
WDI	7.689 ± 4.254	7.36 ± 4.149	0.472^b^
STI	16.83 ± 8.173	16.70 ± 7.959	0.962^b^
*PC (pillows closed position)*			
FHI	0.93 ± 0.912	0.84 ± 0.142	0.983^b^
WDI	7.44 ± 3.967	7.312 ± 3.690	0.982^b^
STI	21.74 ± 10.710	22.23 ± 11.391	0.957^b^
*HR (head right position)*			
FHI	0.85 ± 0.135	0.86 ± 0.116	0.873^b^
WDI	6.32 ± 3.870	6.33 ± 4.048	0.720^b^
STI	21.97 ± 11.341	22.70 ± 11.156	0.343^b^
*HL (head left position)*			
FHI	0.84 ± 0.145	0.83 ± 0.177	0.949^b^
WDI	6.37 ± 4.533	6.44 ± 3.995	0.683^b^
STI	23.84 ± 10.960	23.86 ± 12.515	0.656^b^
*HB (head back position)*			
FHI	0.81 ± 0.159	0.83 ± 0.161	0.214^b^
WDI	6.48 ± 3.654	6.92 ± 4.159	0.324^b^
STI	22.38 ± 11.620	24.26 ± 13.783	0.118^b^
*HF (head forward position)*			
FHI	0.85 ± 0.137	2.05 ± 10.54	0.828^b^
WDI	5.04 ± 3.312	6.01 ± 3.613	0.135^b^
STI	22.35 ± 10.066	23.32 ± 11.801	0.420^b^

^b^Wilcoxon test; ^*∗*^Statistically significant (*p* < 0.0062). FHI, Fourier harmony index; WDI, weight distribution index; STI, stability index. NO (normal open position: standing straight with eyes open); NC (normal closed position: standing straight with eyes closed); PO (pillows open: standing on pillows, with eyes open); PC (pillows closed: standing on pillows, with eyes closed); HR (head right: standing with the head turned right and eyes closed); HL (head left: standing with the head turned left and eyes closed); HB (head back: standing with tilted backward at a 30-degreee angle, with eyes closed); HF (head forward: standing with head tilted forward about 30°, with eyes closed).

## Data Availability

Readers can access the data supporting the conclusions of the study. The data used to support the findings of this study are included within the article.

## Additional Points

*Précis*. Significant changes were not found in patients undergoing dermatochalasis surgery during their evaluation with balance function parameters. Balance function evaluation may provide new approaches to dermatochalasis surgery.
